# 4,4′,4′′-(Methane­triyl)triphenyl tris­(2,2,5,5-tetra­methyl-1-oxyl-3-pyrroline-3-carboxyl­ate) benzene tris­olvate

**DOI:** 10.1107/S1600536810007294

**Published:** 2010-03-03

**Authors:** Denise Schuetz, Dominik Margraf, Thomas F. Prisner, Jan W. Bats

**Affiliations:** a Institut für Physikalische und Theoretische Chemie, Universität Frankfurt, Max-von-Laue-Strasse 7, D-60438 Frankfurt am Main, Germany; b Institut für Organische Chemie, Universität Frankfurt, Max-von-Laue-Strasse 7, D-60438 Frankfurt am Main, Germany

## Abstract

In the asymmetric unit of the title compound, C_46_H_52_N_3_O_9_·3C_6_H_6_, two of the benzene solvent mol­ecules are located in general positions and two are disposed about inversion centers. One of the benzene mol­ecules on an inversion center was grossly disordered and was excluded using the SQUEEZE subroutine in *PLATON* [Spek (2009 ▶). *Acta Cryst*. D**65**, 148–155]. In addition, one of the 2,2,5,5-tetra­methyl-1-oxyl-3-pyrrolin-3-ylcarbonyl groups is disordered over two orientations with refined occupancies of 0.506 (2) and 0.494 (2). The 1-oxyl-3-pyrroline-3-carboxyl­ate groups are essentially planar, with mean deviations from the planes of 0.026 (2), 0.012 (2), 0.034 (4) and 0.011 (4) Å. In the crystal structure, mol­ecules are connected by five weak inter­molecular C—H⋯O and four weak inter­molecular C—H⋯π(benzene) inter­actions.

## Related literature

For the preparation of the title compound see: Godt *et al.* (2000 ▶). For a related structure, see: Margraf *et al.* (2009 ▶). For the treatment of the disordered solvent, see: Spek (2009 ▶).
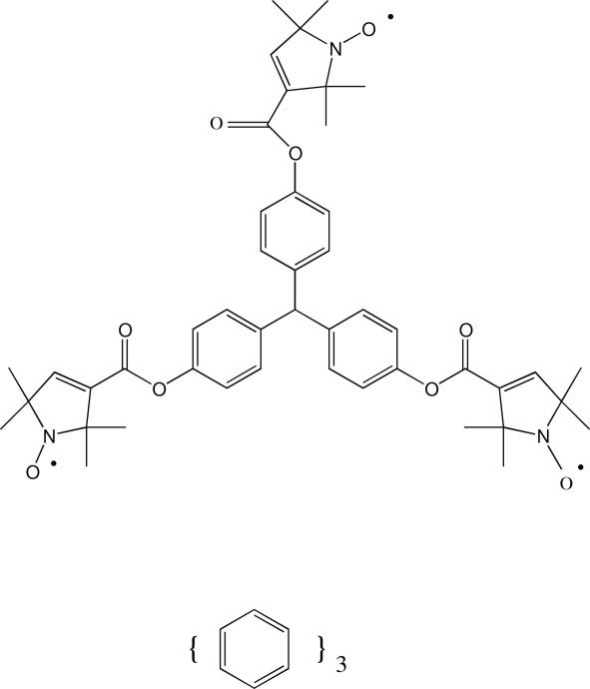


## Experimental

### 

#### Crystal data

C_46_H_52_N_3_O_9_·3C_6_H_6_
*M* *_r_* = 1025.23
Triclinic, 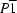

*a* = 10.0810 (13) Å
*b* = 11.7372 (16) Å
*c* = 26.241 (4) Å
α = 98.324 (10)°
β = 92.765 (11)°
γ = 105.308 (10)°
*V* = 2951.1 (7) Å^3^
*Z* = 2
Mo *K*α radiation
μ = 0.08 mm^−1^
*T* = 164 K
0.70 × 0.20 × 0.20 mm

#### Data collection

Siemens SMART 1K CCD diffractometer
Absorption correction: multi-scan (*SADABS*; Sheldrick, 2000 ▶) *T*_min_ = 0.878, *T*_max_ = 0.985
43135 measured reflections
12901 independent reflections
6172 reflections with *I* > 2σ(*I*)
*R*_int_ = 0.054

#### Refinement

*R*[*F* ^2^ > 2σ(*F* ^2^)] = 0.072
*wR*(*F* ^2^) = 0.194
*S* = 1.04
12901 reflections
667 parameters
H-atom parameters constrained
Δρ_max_ = 0.41 e Å^−3^
Δρ_min_ = −0.40 e Å^−3^

### 

Data collection: *SMART* (Siemens, 1995 ▶); cell refinement: *SMART*; data reduction: *SAINT* (Siemens, 1995 ▶); program(s) used to solve structure: *SHELXS97* (Sheldrick, 2008 ▶); program(s) used to refine structure: *SHELXL97* (Sheldrick, 2008 ▶); molecular graphics: *SHELXTL* (Sheldrick, 2008 ▶); software used to prepare material for publication: *SHELXL97*.

## Supplementary Material

Crystal structure: contains datablocks global, I. DOI: 10.1107/S1600536810007294/lh5001sup1.cif

Structure factors: contains datablocks I. DOI: 10.1107/S1600536810007294/lh5001Isup2.hkl

Additional supplementary materials:  crystallographic information; 3D view; checkCIF report

## Figures and Tables

**Table 1:** Hydrogen-bond geometry (Å, °) *Cg*1, *Cg*2, and *Cg*3 are the centroids of the C47–C52, C17–C22 and C56–C61 rings, respectively.

*D*—H⋯*A*	*D*—H	H⋯*A*	*D*⋯*A*	*D*—H⋯*A*
C1—H1*A*⋯O6^i^	1.00	2.49	3.448 (3)	160
C4—H4*A*⋯O9′^ii^	0.95	2.44	3.165 (5)	133
C25—H25*A*⋯O2^iii^	0.95	2.57	3.358 (4)	141
C37—H37*A*⋯O6^i^	0.95	2.57	3.442 (3)	153
C57—H57*A*⋯O6^iv^	0.95	2.58	3.514 (8)	168
C10—H10*A*⋯*Cg*1	0.95	2.84	3.752 (3)	161
C12—H12*A*⋯*Cg*2^iv^	0.98	2.94	3.726 (3)	138
C19—H19*A*⋯*Cg*1^v^	0.95	2.79	3.725 (3)	169
C34—H34*A*⋯*Cg*3	0.95	2.78	3.547 (4)	138

Symmetry codes: (i) 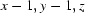; (ii) 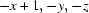; (iii) 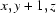; (iv) 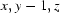; (v) 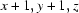.

**Fig. 1.**
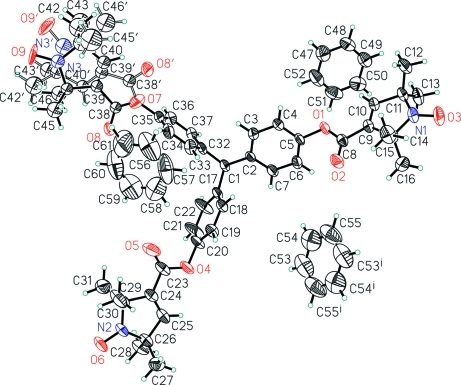
The asymmetric unit of the title compound shown with 50% probability displacement ellipsoids. The H atoms are drawn as small spheres of arbitrary radius. Symmetry equivalent atoms are related by i: 2-x, 1-y, 1-z. A disorder component is shown with open bonds.

**Fig. 2.**
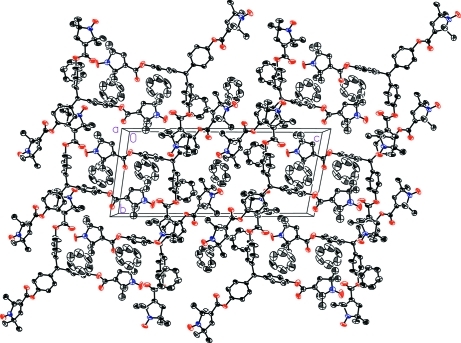
The crystal packing of the title compound, viewed down the a axis. H atoms have been omitted. Along the b-direction adjacent molecules are shown with alternating orientations of the disordered side-chain.

## supplementary crystallographic information

### Comment

The title compound was prepared as a reference compound for pulsed electron-electron double resonance measurements (Godt *et al.*, 2000).

The molecular structure is shown in Fig. 1. The 2,2,5,5-tetramethyl-1-oxyl-3-pyrroline-3-carbonyl group attached to atom O7 is disordered over two orientations with equal occupancies. Short intermolecular contact distances [O8···O8(at 1-x,1-y,-z)=2.371 (5)Å and O8'···O8'(at 1-x,-y,-z)=2.354 (5) Å] show that adjacent groups should occupy alternating orientations along the b-direction, resulting in space group symmetry P 1 with Z'=2. A refinement as an ordered structure in P 1, however, resulted in much higher R-values, showing that the packing must be randomly disordered along at least one other direction.

Each of the 1-oxyl-3-pyrroline groups is approximately planar [mean deviation from plane: 0.026 (2), 0.012 (2), 0.034 (4) and 0.011 (4)Å respectively] and is coplanar with the carbonyloxy group to which it is bonded [torsion angles O1—C8—C9—C10: -0.7 (3)° , O4—C23—C24—C25: 2.4 (4)° , O7—C38—C39—C40: -4.5 (7)° and O7—C38'-C39'-C40': 10.5 (9)° ]. Planarity of this group also has been observed in a related crystal structure (Margraf *et al.*, 2009). The acetoxyphenyl groups are non-planar [torsion angles: C8—O1—C5—C6=-68.2 (3)° , C23—O4—C20—C19=86.6 (3)° , C38—O7—C35—C34=-78.9 (5)° and C38'-O7—C35—C34=67.7 (5)° .

The crystal packing is shown in Fig. 2. The asymmetric unit contains a triphenylmethane-4,4',4"-triyl tris(2,2,5,5-tetramethyl-1-oxyl-3-pyrroline-3-carboxylate) molecule, two benzene solvent molecules in general positions and two benzene solvent molecules positioned about inversion centers. One of the latter groups was found to be seriously disordered and was included in the calculations by using program PLATON/SQUEEZE (Spek, 2009). There are five intermolecular C—H···O contacts with H···O distances between 2.44 and 2.58 Å and four intermolecular C—H···π_benzene_ contacts with H···Cg distances between 2.78 and 2.94 Å (Table 1, Cg represents the centroid of the benzene ring).

### Experimental

The title compound was prepared from tris(4-hydroxyphenyl)methane in analogy to the procedure described by Godt *et al.* (2000). Single crystals were obtained by recrystallization of the compound from benzene.

### Refinement

The H atoms were positioned geometrically and treated as riding: C_primary_—H=1.00 Å, C_methyl_—H=0.98 Å, C_planar_—H=0.95 Å, U_iso_(*H*)=1.2U_eq_(C_non-methyl_) and U_iso_(*H*)=1.5U_eq_(C_methyl_). A benzene solvent molecule seriously disordered about the inversion center at (0, 0, 0) was accounted for by using the program PLATON/SQUEEZE (Spek, 2009). The electron density count in this region was found to be 42 electrons, in perfect agreement with the value expected for a benzene molecule and the contribution from the additional benzene molecule was added to the empirical formula. The 2,2,5,5-tetramethyl-1-oxyl-3-pyrroline-3-carbonyl group attached to atom O7 was found to be disordered over two possible orientations. Consequently, atoms in this group were refined as split atoms and treated as isotropic, except for the O atoms which were refined with anisotropic displacement parameters. The occupancy factor refined to 0.494 (2) for atoms, O8, O9, N3 and C38 → C46 and to 0.506 (2) for atoms O8', O9', N3' and C38' → C46'. A refinement as an ordered structure in space group P 1 converged at wR(F^2^)=0.295, R[F^2^>2σ(F^2^)]=0.107 and showed residual density up to 1.21 e.A^-3^ in the 2,2,5,5-tetramethyl-1-oxyl-3-pyrroline-3-carbonyl group, which was disordered in space group P 1. Thus an ordered structure can be excluded. Oxygen atom O7 could not be split to meaningful positions within the experimental resolution. As a result, the observed values of the C35-O7-C38 and C35-O7-C38' angles [128.4 (3) and 140.5 (3)° ] are too large.

### Crystal data

**Table:**

C_46_H_52_N_3_O_9_·3C_6_H_6_	*Z* = 2
*M**_r_* = 1025.23	*F*(000) = 1094
Triclinic, *P*1	*D*_x_ = 1.154 Mg m^−^^3^
Hall symbol: -P 1	Mo *K*α radiation, λ = 0.71073 Å
*a* = 10.0810 (13) Å	Cell parameters from 204 reflections
*b* = 11.7372 (16) Å	θ = 3–23°
*c* = 26.241 (4) Å	µ = 0.08 mm^−^^1^
α = 98.324 (10)°	*T* = 164 K
β = 92.765 (11)°	Rod, yellow
γ = 105.308 (10)°	0.70 × 0.20 × 0.20 mm
*V* = 2951.1 (7) Å^3^	

### Data collection

**Table:**

Siemens SMART 1K CCD diffractometer	12901 independent reflections
Radiation source: normal-focus sealed tube	6172 reflections with *I* > 2σ(*I*)
graphite	*R*_int_ = 0.054
ω scans	θ_max_ = 27.5°, θ_min_ = 1.6°
Absorption correction: multi-scan (*SADABS*; Sheldrick, 2000)	*h* = −13→13
*T*_min_ = 0.878, *T*_max_ = 0.985	*k* = −14→15
43135 measured reflections	*l* = −32→33

### Refinement

**Table:**

Refinement on *F*^2^	Primary atom site location: structure-invariant direct methods
Least-squares matrix: full	Secondary atom site location: difference Fourier map
*R*[*F*^2^ > 2σ(*F*^2^)] = 0.072	Hydrogen site location: inferred from neighbouring sites
*wR*(*F*^2^) = 0.194	H-atom parameters constrained
*S* = 1.04	*w* = 1/[σ^2^(*F*_o_^2^) + (0.09*P*)^2^] where *P* = (*F*_o_^2^ + 2*F*_c_^2^)/3
12901 reflections	(Δ/σ)_max_ = 0.002
667 parameters	Δρ_max_ = 0.41 e Å^−^^3^
0 restraints	Δρ_min_ = −0.40 e Å^−^^3^

### Special details

**Table:**

Geometry. All esds (except the esd in the dihedral angle between two l.s. planes) are estimated using the full covariance matrix. The cell esds are taken into account individually in the estimation of esds in distances, angles and torsion angles; correlations between esds in cell parameters are only used when they are defined by crystal symmetry. An approximate (isotropic) treatment of cell esds is used for estimating esds involving l.s. planes.
Refinement. Refinement of *F*^2^ against ALL reflections. The weighted *R*-factor wR and goodness of fit *S* are based on *F*^2^, conventional *R*-factors *R* are based on *F*, with *F* set to zero for negative *F*^2^. The threshold expression of *F*^2^ > σ(*F*^2^) is used only for calculating *R*-factors(gt) etc. and is not relevant to the choice of reflections for refinement. *R*-factors based on *F*^2^ are statistically about twice as large as those based on *F*, and *R*- factors based on ALL data will be even larger.

### Fractional atomic coordinates and isotropic or equivalent isotropic displacement parameters (Å^2^)

**Table:**

	*x*	*y*	*z*	*U*_iso_*/*U*_eq_	Occ. (<1)
O1	0.53115 (18)	−0.01253 (13)	0.36733 (6)	0.0452 (4)	
O2	0.72796 (17)	0.08878 (15)	0.41627 (7)	0.0503 (5)	
O3	0.6843 (2)	−0.28486 (17)	0.51844 (8)	0.0653 (6)	
O4	0.81097 (19)	0.83869 (14)	0.30718 (7)	0.0582 (5)	
O5	0.8868 (2)	0.81827 (17)	0.22723 (9)	0.0745 (6)	
O6	1.12533 (19)	1.29224 (14)	0.25223 (7)	0.0590 (5)	
O7	0.4972 (2)	0.25906 (19)	0.03656 (8)	0.0798 (7)	
O8	0.5327 (5)	0.4303 (3)	0.02292 (14)	0.0667 (13)	0.494 (2)
O9	0.5887 (5)	0.2649 (4)	−0.17679 (13)	0.0756 (15)	0.494 (2)
O8'	0.5441 (5)	0.1012 (3)	0.01896 (14)	0.0781 (14)	0.506 (2)
O9'	0.5181 (7)	0.1270 (5)	−0.18702 (15)	0.122 (2)	0.506 (2)
N1	0.6415 (2)	−0.24001 (18)	0.48143 (8)	0.0458 (5)	
N2	1.0619 (2)	1.20024 (17)	0.27055 (8)	0.0469 (6)	
N3	0.5720 (5)	0.2458 (5)	−0.13101 (17)	0.0466 (12)*	0.494 (2)
N3'	0.5159 (6)	0.1762 (6)	−0.1401 (2)	0.0668 (15)*	0.506 (2)
C1	0.4798 (2)	0.35575 (19)	0.25423 (9)	0.0358 (6)	
H1A	0.3826	0.3572	0.2595	0.043*	
C2	0.5085 (2)	0.26105 (19)	0.28438 (9)	0.0327 (5)	
C3	0.5311 (3)	0.1558 (2)	0.26070 (9)	0.0453 (6)	
H3A	0.5387	0.1441	0.2245	0.054*	
C4	0.5429 (3)	0.0661 (2)	0.28895 (9)	0.0437 (6)	
H4A	0.5576	−0.0063	0.2722	0.052*	
C5	0.5329 (2)	0.08435 (19)	0.34088 (9)	0.0381 (6)	
C6	0.5110 (3)	0.1883 (2)	0.36585 (10)	0.0483 (7)	
H6A	0.5029	0.1996	0.4020	0.058*	
C7	0.5011 (3)	0.2761 (2)	0.33715 (10)	0.0452 (7)	
H7A	0.4887	0.3491	0.3543	0.054*	
C8	0.6331 (3)	−0.0003 (2)	0.40459 (9)	0.0367 (6)	
C9	0.6121 (2)	−0.1091 (2)	0.42854 (9)	0.0348 (6)	
C10	0.5068 (2)	−0.2069 (2)	0.41613 (9)	0.0372 (6)	
H10A	0.4356	−0.2158	0.3896	0.045*	
C11	0.5119 (3)	−0.3016 (2)	0.44818 (10)	0.0426 (6)	
C12	0.5255 (3)	−0.4162 (2)	0.41644 (11)	0.0627 (8)	
H12A	0.6038	−0.3974	0.3955	0.094*	
H12B	0.4406	−0.4545	0.3937	0.094*	
H12C	0.5407	−0.4708	0.4397	0.094*	
C13	0.3917 (3)	−0.3261 (3)	0.48212 (11)	0.0567 (8)	
H13A	0.3862	−0.2505	0.5021	0.085*	
H13B	0.4066	−0.3798	0.5058	0.085*	
H13C	0.3053	−0.3638	0.4602	0.085*	
C14	0.7124 (2)	−0.1188 (2)	0.47093 (9)	0.0409 (6)	
C15	0.8546 (3)	−0.1184 (3)	0.45284 (12)	0.0599 (8)	
H15A	0.8436	−0.1776	0.4214	0.090*	
H15B	0.9102	−0.1388	0.4800	0.090*	
H15C	0.9011	−0.0387	0.4456	0.090*	
C16	0.7252 (3)	−0.0275 (3)	0.51980 (10)	0.0613 (8)	
H16A	0.6329	−0.0289	0.5303	0.092*	
H16B	0.7717	0.0526	0.5130	0.092*	
H16C	0.7791	−0.0476	0.5475	0.092*	
C17	0.5699 (2)	0.4829 (2)	0.27361 (9)	0.0381 (6)	
C18	0.7073 (2)	0.5096 (2)	0.29182 (9)	0.0398 (6)	
H18A	0.7467	0.4464	0.2967	0.048*	
C19	0.7888 (3)	0.6274 (2)	0.30317 (10)	0.0444 (6)	
H19A	0.8833	0.6450	0.3155	0.053*	
C20	0.7306 (3)	0.7174 (2)	0.29620 (11)	0.0516 (7)	
C21	0.5926 (3)	0.6943 (2)	0.28051 (15)	0.0825 (11)	
H21A	0.5525	0.7580	0.2776	0.099*	
C22	0.5140 (3)	0.5773 (2)	0.26915 (14)	0.0728 (10)	
H22A	0.4188	0.5606	0.2580	0.087*	
C23	0.8817 (3)	0.8800 (2)	0.26730 (13)	0.0531 (7)	
C24	0.9470 (3)	1.0102 (2)	0.27938 (11)	0.0443 (6)	
C25	0.9442 (3)	1.0799 (2)	0.32329 (11)	0.0492 (7)	
H25A	0.9021	1.0508	0.3522	0.059*	
C26	1.0145 (3)	1.2103 (2)	0.32310 (10)	0.0470 (7)	
C27	1.1365 (3)	1.2618 (3)	0.36340 (11)	0.0623 (8)	
H27A	1.2006	1.2119	0.3597	0.093*	
H27B	1.1039	1.2636	0.3980	0.093*	
H27C	1.1840	1.3434	0.3586	0.093*	
C28	0.9105 (3)	1.2851 (2)	0.32690 (13)	0.0644 (8)	
H28A	0.8336	1.2492	0.3002	0.097*	
H28B	0.9562	1.3669	0.3218	0.097*	
H28C	0.8754	1.2870	0.3611	0.097*	
C29	1.0226 (3)	1.0784 (2)	0.24016 (10)	0.0535 (7)	
C30	1.1532 (4)	1.0425 (2)	0.22696 (14)	0.0836 (11)	
H30A	1.2097	1.0462	0.2589	0.125*	
H30B	1.2059	1.0974	0.2058	0.125*	
H30C	1.1279	0.9606	0.2077	0.125*	
C31	0.9301 (5)	1.0754 (3)	0.19274 (13)	0.1072 (15)	
H31A	0.8484	1.0998	0.2033	0.161*	
H31B	0.9013	0.9940	0.1730	0.161*	
H31C	0.9805	1.1306	0.1711	0.161*	
C32	0.4854 (3)	0.3272 (2)	0.19600 (9)	0.0384 (6)	
C33	0.6103 (3)	0.3336 (2)	0.17411 (10)	0.0512 (7)	
H33A	0.6937	0.3539	0.1960	0.061*	
C34	0.6145 (3)	0.3109 (2)	0.12123 (11)	0.0534 (7)	
H34A	0.7000	0.3162	0.1067	0.064*	
C35	0.4927 (3)	0.2805 (2)	0.08983 (9)	0.0479 (7)	
C36	0.3685 (3)	0.2738 (2)	0.10991 (10)	0.0508 (7)	
H36A	0.2856	0.2534	0.0877	0.061*	
C37	0.3655 (3)	0.2974 (2)	0.16310 (10)	0.0435 (6)	
H37A	0.2796	0.2930	0.1772	0.052*	
C38	0.5259 (5)	0.3267 (5)	0.00924 (18)	0.0342 (11)*	0.494 (2)
C39	0.5383 (5)	0.2737 (6)	−0.0439 (2)	0.0372 (13)*	0.494 (2)
C40	0.5269 (6)	0.1600 (6)	−0.0584 (3)	0.0435 (19)*	0.494 (2)
H40A	0.5123	0.1041	−0.0350	0.052*	0.494 (2)
C41	0.5385 (8)	0.1254 (7)	−0.1142 (3)	0.062 (2)*	0.494 (2)
C42	0.6402 (8)	0.0620 (7)	−0.1295 (3)	0.088 (2)*	0.494 (2)
H42A	0.7313	0.1072	−0.1123	0.131*	0.494 (2)
H42B	0.6438	0.0537	−0.1671	0.131*	0.494 (2)
H42C	0.6141	−0.0176	−0.1194	0.131*	0.494 (2)
C43	0.3930 (7)	0.0497 (7)	−0.1432 (3)	0.088 (2)*	0.494 (2)
H43A	0.4031	0.0283	−0.1800	0.132*	0.494 (2)
H43B	0.3267	0.0975	−0.1391	0.132*	0.494 (2)
H43C	0.3595	−0.0234	−0.1282	0.132*	0.494 (2)
C44	0.5595 (5)	0.3435 (5)	−0.0885 (2)	0.0382 (12)*	0.494 (2)
C45	0.6952 (6)	0.4419 (5)	−0.0809 (2)	0.0639 (17)*	0.494 (2)
H45A	0.7710	0.4084	−0.0721	0.096*	0.494 (2)
H45B	0.6908	0.5052	−0.0528	0.096*	0.494 (2)
H45C	0.7110	0.4754	−0.1129	0.096*	0.494 (2)
C46	0.4390 (6)	0.3926 (5)	−0.1013 (2)	0.0616 (17)*	0.494 (2)
H46A	0.3529	0.3279	−0.1056	0.092*	0.494 (2)
H46B	0.4521	0.4257	−0.1335	0.092*	0.494 (2)
H46C	0.4345	0.4557	−0.0731	0.092*	0.494 (2)
C38'	0.5238 (5)	0.1855 (5)	0.00195 (19)	0.0417 (13)*	0.506 (2)
C39'	0.5230 (6)	0.2059 (8)	−0.0516 (2)	0.0424 (16)*	0.506 (2)
C40'	0.5158 (5)	0.3052 (5)	−0.0676 (2)	0.0384 (12)*	0.506 (2)
H40B	0.5116	0.3748	−0.0449	0.046*	0.506 (2)
C41'	0.5147 (5)	0.2980 (5)	−0.1243 (2)	0.0438 (13)*	0.506 (2)
C42'	0.6415 (7)	0.3871 (6)	−0.1412 (3)	0.082 (2)*	0.506 (2)
H42D	0.7265	0.3733	−0.1269	0.123*	0.506 (2)
H42E	0.6399	0.4693	−0.1282	0.123*	0.506 (2)
H42F	0.6382	0.3750	−0.1790	0.123*	0.506 (2)
C43'	0.3833 (6)	0.3192 (5)	−0.1471 (2)	0.0643 (17)*	0.506 (2)
H43D	0.3028	0.2629	−0.1366	0.096*	0.506 (2)
H43E	0.3810	0.3068	−0.1849	0.096*	0.506 (2)
H43F	0.3814	0.4014	−0.1343	0.096*	0.506 (2)
C44'	0.5244 (8)	0.1077 (7)	−0.0966 (4)	0.071 (3)*	0.506 (2)
C45'	0.6756 (9)	0.0850 (9)	−0.0961 (4)	0.119 (3)*	0.506 (2)
H45D	0.7463	0.1620	−0.0920	0.178*	0.506 (2)
H45E	0.6832	0.0357	−0.1287	0.178*	0.506 (2)
H45F	0.6891	0.0435	−0.0672	0.178*	0.506 (2)
C46'	0.4074 (8)	−0.0006 (6)	−0.1012 (3)	0.092 (2)*	0.506 (2)
H46D	0.3208	0.0223	−0.1002	0.139*	0.506 (2)
H46E	0.4172	−0.0432	−0.0723	0.139*	0.506 (2)
H46F	0.4065	−0.0529	−0.1339	0.139*	0.506 (2)
C47	0.2098 (3)	−0.3127 (3)	0.28489 (12)	0.0630 (8)	
H47A	0.2370	−0.3313	0.2512	0.076*	
C48	0.1703 (3)	−0.4011 (2)	0.31486 (12)	0.0572 (8)	
H48A	0.1703	−0.4810	0.3018	0.069*	
C49	0.1310 (3)	−0.3743 (3)	0.36369 (12)	0.0563 (7)	
H49A	0.1036	−0.4355	0.3842	0.068*	
C50	0.1314 (3)	−0.2589 (3)	0.38264 (13)	0.0654 (8)	
H50A	0.1049	−0.2404	0.4164	0.078*	
C51	0.1698 (3)	−0.1704 (3)	0.35336 (16)	0.0736 (10)	
H51A	0.1693	−0.0908	0.3666	0.088*	
C52	0.2094 (3)	−0.1976 (3)	0.30418 (16)	0.0753 (10)	
H52A	0.2364	−0.1363	0.2837	0.090*	
C53	0.9261 (6)	0.5700 (5)	0.4825 (2)	0.1024 (15)	
H53A	0.8736	0.6184	0.4701	0.123*	
C54	0.8921 (4)	0.4510 (6)	0.46383 (17)	0.0996 (13)	
H54A	0.8164	0.4167	0.4383	0.119*	
C55	0.9666 (7)	0.3799 (4)	0.4815 (2)	0.1074 (17)	
H55A	0.9427	0.2965	0.4685	0.129*	
C56	0.9982 (5)	0.3708 (5)	0.1088 (3)	0.130 (2)	
H56A	1.0264	0.2992	0.1037	0.155*	
C57	1.0004 (4)	0.4293 (7)	0.1570 (3)	0.118 (2)	
H57A	1.0253	0.3970	0.1860	0.142*	
C58	0.9666 (4)	0.5348 (6)	0.1639 (2)	0.1088 (15)	
H58A	0.9696	0.5765	0.1980	0.131*	
C59	0.9297 (5)	0.5806 (5)	0.1240 (3)	0.1202 (17)	
H59A	0.9081	0.6552	0.1295	0.144*	
C60	0.9229 (6)	0.5220 (7)	0.0763 (3)	0.137 (2)	
H60A	0.8950	0.5553	0.0481	0.164*	
C61	0.9551 (6)	0.4146 (6)	0.0666 (2)	0.143 (2)	
H61A	0.9481	0.3723	0.0324	0.171*	

### Atomic displacement parameters (Å^2^)

**Table:**

	*U*^11^	*U*^22^	*U*^33^	*U*^12^	*U*^13^	*U*^23^
O1	0.0549 (11)	0.0294 (9)	0.0482 (10)	0.0016 (8)	−0.0070 (9)	0.0187 (8)
O2	0.0420 (11)	0.0401 (11)	0.0661 (12)	0.0012 (9)	0.0022 (9)	0.0196 (9)
O3	0.0700 (13)	0.0698 (13)	0.0685 (13)	0.0271 (11)	−0.0038 (10)	0.0403 (11)
O4	0.0603 (12)	0.0294 (10)	0.0776 (14)	−0.0058 (9)	0.0028 (10)	0.0202 (9)
O5	0.0835 (16)	0.0350 (11)	0.0957 (17)	0.0015 (10)	0.0256 (13)	0.0019 (11)
O6	0.0695 (13)	0.0312 (10)	0.0778 (13)	0.0054 (9)	0.0229 (10)	0.0241 (9)
O7	0.1142 (19)	0.0721 (15)	0.0448 (13)	0.0173 (13)	0.0237 (12)	−0.0077 (11)
O8	0.126 (4)	0.039 (2)	0.044 (2)	0.033 (2)	0.021 (2)	0.0153 (17)
O9	0.114 (4)	0.114 (4)	0.023 (2)	0.054 (3)	0.025 (2)	0.039 (2)
O8'	0.157 (4)	0.048 (2)	0.046 (2)	0.051 (3)	0.012 (2)	0.0189 (18)
O9'	0.267 (7)	0.102 (4)	0.031 (2)	0.110 (5)	0.033 (3)	0.003 (2)
N1	0.0466 (13)	0.0473 (13)	0.0520 (14)	0.0189 (11)	0.0046 (11)	0.0249 (11)
N2	0.0520 (14)	0.0282 (12)	0.0610 (15)	0.0057 (10)	0.0105 (11)	0.0166 (10)
C1	0.0383 (14)	0.0304 (13)	0.0405 (14)	0.0073 (11)	0.0042 (11)	0.0155 (10)
C2	0.0345 (13)	0.0236 (12)	0.0376 (14)	0.0009 (10)	0.0013 (10)	0.0114 (10)
C3	0.0679 (18)	0.0353 (15)	0.0350 (14)	0.0160 (13)	0.0039 (13)	0.0104 (11)
C4	0.0641 (18)	0.0284 (13)	0.0414 (16)	0.0151 (12)	0.0035 (13)	0.0103 (11)
C5	0.0462 (15)	0.0244 (13)	0.0426 (15)	0.0025 (11)	−0.0002 (12)	0.0164 (11)
C6	0.0709 (19)	0.0384 (15)	0.0399 (15)	0.0151 (13)	0.0148 (13)	0.0169 (12)
C7	0.0634 (18)	0.0296 (14)	0.0455 (16)	0.0131 (12)	0.0140 (13)	0.0120 (11)
C8	0.0374 (15)	0.0338 (14)	0.0413 (15)	0.0085 (12)	0.0116 (12)	0.0138 (11)
C9	0.0358 (14)	0.0347 (14)	0.0392 (14)	0.0133 (11)	0.0097 (11)	0.0144 (11)
C10	0.0386 (14)	0.0372 (14)	0.0394 (14)	0.0105 (12)	0.0048 (11)	0.0167 (11)
C11	0.0476 (16)	0.0340 (14)	0.0510 (16)	0.0117 (12)	0.0058 (13)	0.0210 (12)
C12	0.086 (2)	0.0397 (16)	0.069 (2)	0.0240 (15)	0.0102 (17)	0.0195 (14)
C13	0.0541 (18)	0.0594 (18)	0.0647 (19)	0.0140 (14)	0.0143 (14)	0.0363 (15)
C14	0.0412 (15)	0.0409 (15)	0.0449 (15)	0.0150 (12)	0.0024 (12)	0.0146 (12)
C15	0.0400 (16)	0.066 (2)	0.080 (2)	0.0177 (14)	0.0050 (15)	0.0270 (16)
C16	0.076 (2)	0.0577 (19)	0.0503 (18)	0.0206 (16)	−0.0059 (15)	0.0069 (14)
C17	0.0392 (15)	0.0290 (13)	0.0473 (15)	0.0054 (11)	0.0017 (12)	0.0184 (11)
C18	0.0418 (16)	0.0321 (14)	0.0496 (15)	0.0128 (12)	0.0045 (12)	0.0156 (11)
C19	0.0373 (15)	0.0381 (15)	0.0586 (17)	0.0074 (12)	0.0060 (12)	0.0153 (12)
C20	0.0487 (18)	0.0263 (14)	0.077 (2)	−0.0002 (12)	0.0014 (14)	0.0205 (13)
C21	0.064 (2)	0.0321 (17)	0.151 (3)	0.0093 (15)	−0.027 (2)	0.0321 (18)
C22	0.0427 (17)	0.0339 (16)	0.142 (3)	0.0057 (13)	−0.0173 (18)	0.0319 (17)
C23	0.0465 (17)	0.0308 (15)	0.082 (2)	0.0053 (13)	0.0054 (16)	0.0195 (15)
C24	0.0418 (15)	0.0304 (14)	0.0622 (18)	0.0090 (12)	0.0054 (13)	0.0137 (13)
C25	0.0439 (16)	0.0367 (15)	0.071 (2)	0.0089 (12)	0.0178 (14)	0.0227 (14)
C26	0.0436 (16)	0.0330 (14)	0.0651 (18)	0.0063 (12)	0.0166 (14)	0.0143 (12)
C27	0.0563 (19)	0.0573 (19)	0.066 (2)	0.0076 (15)	−0.0022 (16)	0.0045 (15)
C28	0.0548 (18)	0.0371 (16)	0.103 (2)	0.0112 (14)	0.0236 (17)	0.0133 (15)
C29	0.078 (2)	0.0296 (15)	0.0514 (17)	0.0084 (14)	0.0068 (15)	0.0124 (12)
C30	0.115 (3)	0.0370 (17)	0.106 (3)	0.0186 (17)	0.070 (2)	0.0210 (16)
C31	0.178 (4)	0.051 (2)	0.074 (2)	0.003 (2)	−0.039 (3)	0.0182 (17)
C32	0.0457 (16)	0.0292 (13)	0.0434 (15)	0.0080 (11)	0.0057 (13)	0.0195 (11)
C33	0.0536 (18)	0.0550 (17)	0.0491 (17)	0.0151 (14)	0.0057 (14)	0.0210 (13)
C34	0.066 (2)	0.0510 (17)	0.0520 (18)	0.0192 (15)	0.0219 (16)	0.0239 (13)
C35	0.084 (2)	0.0296 (14)	0.0330 (15)	0.0169 (14)	0.0055 (15)	0.0118 (11)
C36	0.0592 (19)	0.0459 (16)	0.0475 (17)	0.0124 (14)	0.0038 (14)	0.0129 (13)
C37	0.0508 (17)	0.0393 (15)	0.0421 (16)	0.0095 (12)	0.0064 (13)	0.0162 (11)
C47	0.0531 (19)	0.065 (2)	0.071 (2)	0.0108 (16)	0.0041 (15)	0.0203 (17)
C48	0.0491 (18)	0.0398 (16)	0.080 (2)	0.0088 (13)	0.0016 (16)	0.0097 (15)
C49	0.0403 (16)	0.0519 (19)	0.075 (2)	0.0052 (13)	0.0040 (15)	0.0204 (15)
C50	0.0527 (19)	0.059 (2)	0.083 (2)	0.0197 (16)	−0.0031 (16)	0.0030 (18)
C51	0.065 (2)	0.047 (2)	0.105 (3)	0.0186 (16)	−0.021 (2)	0.0022 (19)
C52	0.065 (2)	0.053 (2)	0.105 (3)	0.0042 (17)	−0.021 (2)	0.035 (2)
C53	0.119 (4)	0.090 (4)	0.130 (4)	0.057 (3)	0.074 (3)	0.046 (3)
C54	0.079 (3)	0.114 (4)	0.107 (3)	0.016 (3)	0.051 (2)	0.028 (3)
C55	0.134 (4)	0.061 (3)	0.132 (4)	0.020 (3)	0.085 (4)	0.021 (3)
C56	0.112 (4)	0.121 (4)	0.202 (6)	0.071 (3)	0.083 (4)	0.085 (5)
C57	0.055 (2)	0.177 (6)	0.145 (5)	0.023 (3)	0.019 (3)	0.114 (5)
C58	0.058 (3)	0.158 (5)	0.102 (4)	0.010 (3)	0.011 (2)	0.027 (3)
C59	0.092 (3)	0.126 (4)	0.166 (5)	0.056 (3)	0.031 (4)	0.042 (4)
C60	0.147 (5)	0.157 (6)	0.146 (5)	0.064 (4)	0.040 (4)	0.103 (5)
C61	0.157 (5)	0.170 (6)	0.119 (4)	0.054 (4)	0.089 (4)	0.040 (4)

### Geometric parameters (Å, °)

**Table:**

O1—C8	1.348 (3)	C31—H31A	0.9800
O1—C5	1.412 (3)	C31—H31B	0.9800
O2—C8	1.205 (3)	C31—H31C	0.9800
O3—N1	1.279 (2)	C32—C37	1.385 (3)
O4—C23	1.369 (3)	C32—C33	1.398 (3)
O4—C20	1.419 (3)	C33—C34	1.380 (4)
O5—C23	1.199 (3)	C33—H33A	0.9500
O6—N2	1.273 (2)	C34—C35	1.379 (4)
O7—C38	1.137 (5)	C34—H34A	0.9500
O7—C38'	1.250 (5)	C35—C36	1.369 (4)
O7—C35	1.389 (3)	C36—C37	1.387 (3)
O8—C38	1.200 (5)	C36—H36A	0.9500
O9—N3	1.264 (5)	C37—H37A	0.9500
O8'—C38'	1.203 (6)	C38—C39	1.466 (7)
O9'—N3'	1.285 (6)	C39—C40	1.306 (9)
N1—C11	1.483 (3)	C39—C44	1.516 (8)
N1—C14	1.486 (3)	C40—C41	1.481 (11)
N2—C29	1.476 (3)	C40—H40A	0.9500
N2—C26	1.480 (3)	C41—C42	1.457 (10)
N3—C41	1.499 (10)	C41—C43	1.590 (10)
N3—C44	1.515 (7)	C42—H42A	0.9800
N3'—C41'	1.434 (8)	C42—H42B	0.9800
N3'—C44'	1.500 (10)	C42—H42C	0.9800
C1—C32	1.523 (3)	C43—H43A	0.9800
C1—C17	1.527 (3)	C43—H43B	0.9800
C1—C2	1.532 (3)	C43—H43C	0.9800
C1—H1A	1.0000	C44—C46	1.518 (7)
C2—C7	1.379 (3)	C44—C45	1.522 (8)
C2—C3	1.379 (3)	C45—H45A	0.9800
C3—C4	1.399 (3)	C45—H45B	0.9800
C3—H3A	0.9500	C45—H45C	0.9800
C4—C5	1.361 (3)	C46—H46A	0.9800
C4—H4A	0.9500	C46—H46B	0.9800
C5—C6	1.376 (3)	C46—H46C	0.9800
C6—C7	1.382 (3)	C38'—C39'	1.460 (8)
C6—H6A	0.9500	C39'—C40'	1.313 (9)
C7—H7A	0.9500	C39'—C44'	1.529 (11)
C8—C9	1.474 (3)	C40'—C41'	1.480 (8)
C9—C10	1.329 (3)	C40'—H40B	0.9500
C9—C14	1.504 (3)	C41'—C43'	1.521 (7)
C10—C11	1.498 (3)	C41'—C42'	1.550 (8)
C10—H10A	0.9500	C42'—H42D	0.9800
C11—C12	1.518 (3)	C42'—H42E	0.9800
C11—C13	1.529 (3)	C42'—H42F	0.9800
C12—H12A	0.9800	C43'—H43D	0.9800
C12—H12B	0.9800	C43'—H43E	0.9800
C12—H12C	0.9800	C43'—H43F	0.9800
C13—H13A	0.9800	C44'—C46'	1.474 (10)
C13—H13B	0.9800	C44'—C45'	1.615 (12)
C13—H13C	0.9800	C45'—H45D	0.9800
C14—C16	1.524 (4)	C45'—H45E	0.9800
C14—C15	1.531 (3)	C45'—H45F	0.9800
C15—H15A	0.9800	C46'—H46D	0.9800
C15—H15B	0.9800	C46'—H46E	0.9800
C15—H15C	0.9800	C46'—H46F	0.9800
C16—H16A	0.9800	C47—C52	1.374 (4)
C16—H16B	0.9800	C47—C48	1.379 (4)
C16—H16C	0.9800	C47—H47A	0.9500
C17—C18	1.382 (3)	C48—C49	1.377 (4)
C17—C22	1.385 (3)	C48—H48A	0.9500
C18—C19	1.390 (3)	C49—C50	1.372 (4)
C18—H18A	0.9500	C49—H49A	0.9500
C19—C20	1.367 (3)	C50—C51	1.366 (4)
C19—H19A	0.9500	C50—H50A	0.9500
C20—C21	1.375 (4)	C51—C52	1.387 (5)
C21—C22	1.374 (4)	C51—H51A	0.9500
C21—H21A	0.9500	C52—H52A	0.9500
C22—H22A	0.9500	C53—C55^i^	1.347 (6)
C23—C24	1.475 (4)	C53—C54	1.357 (6)
C24—C25	1.319 (4)	C53—H53A	0.9500
C24—C29	1.508 (3)	C54—C55	1.373 (6)
C25—C26	1.508 (3)	C54—H54A	0.9500
C25—H25A	0.9500	C55—C53^i^	1.347 (6)
C26—C27	1.515 (4)	C55—H55A	0.9500
C26—C28	1.532 (4)	C56—C57	1.346 (7)
C27—H27A	0.9800	C56—C61	1.384 (7)
C27—H27B	0.9800	C56—H56A	0.9500
C27—H27C	0.9800	C57—C58	1.358 (7)
C28—H28A	0.9800	C57—H57A	0.9500
C28—H28B	0.9800	C58—C59	1.326 (6)
C28—H28C	0.9800	C58—H58A	0.9500
C29—C31	1.509 (4)	C59—C60	1.327 (7)
C29—C30	1.525 (4)	C59—H59A	0.9500
C30—H30A	0.9800	C60—C61	1.375 (7)
C30—H30B	0.9800	C60—H60A	0.9500
C30—H30C	0.9800	C61—H61A	0.9500
			
C8—O1—C5	118.87 (18)	C34—C33—C32	121.1 (3)
C23—O4—C20	115.0 (2)	C34—C33—H33A	119.4
C38—O7—C35	128.4 (3)	C32—C33—H33A	119.4
C38'—O7—C35	140.5 (3)	C35—C34—C33	118.9 (3)
O3—N1—C11	122.38 (19)	C35—C34—H34A	120.5
O3—N1—C14	122.5 (2)	C33—C34—H34A	120.5
C11—N1—C14	115.02 (18)	C36—C35—C34	121.5 (2)
O6—N2—C29	122.8 (2)	C36—C35—O7	119.6 (3)
O6—N2—C26	121.55 (19)	C34—C35—O7	118.9 (3)
C29—N2—C26	115.49 (18)	C35—C36—C37	119.1 (3)
O9—N3—C41	125.6 (5)	C35—C36—H36A	120.5
O9—N3—C44	121.5 (5)	C37—C36—H36A	120.5
C41—N3—C44	112.1 (5)	C32—C37—C36	121.2 (2)
O9'—N3'—C41'	125.1 (6)	C32—C37—H37A	119.4
O9'—N3'—C44'	120.0 (6)	C36—C37—H37A	119.4
C41'—N3'—C44'	114.8 (5)	O7—C38—O8	121.0 (5)
C32—C1—C17	108.73 (17)	O7—C38—C39	114.1 (5)
C32—C1—C2	114.44 (18)	O8—C38—C39	124.5 (5)
C17—C1—C2	114.55 (19)	C40—C39—C38	123.7 (6)
C32—C1—H1A	106.1	C40—C39—C44	112.4 (5)
C17—C1—H1A	106.1	C38—C39—C44	123.8 (5)
C2—C1—H1A	106.1	C39—C40—C41	114.6 (6)
C7—C2—C3	117.7 (2)	C39—C40—H40A	122.7
C7—C2—C1	119.1 (2)	C41—C40—H40A	122.7
C3—C2—C1	123.0 (2)	C42—C41—C40	118.7 (8)
C2—C3—C4	121.3 (2)	C42—C41—N3	111.0 (6)
C2—C3—H3A	119.3	C40—C41—N3	100.5 (6)
C4—C3—H3A	119.3	C42—C41—C43	107.8 (6)
C5—C4—C3	118.8 (2)	C40—C41—C43	110.9 (6)
C5—C4—H4A	120.6	N3—C41—C43	107.3 (7)
C3—C4—H4A	120.6	C41—C42—H42A	109.5
C4—C5—C6	121.5 (2)	C41—C42—H42B	109.5
C4—C5—O1	117.3 (2)	H42A—C42—H42B	109.5
C6—C5—O1	120.9 (2)	C41—C42—H42C	109.5
C5—C6—C7	118.6 (2)	H42A—C42—H42C	109.5
C5—C6—H6A	120.7	H42B—C42—H42C	109.5
C7—C6—H6A	120.7	C41—C43—H43A	109.5
C2—C7—C6	122.1 (2)	C41—C43—H43B	109.5
C2—C7—H7A	119.0	H43A—C43—H43B	109.5
C6—C7—H7A	119.0	C41—C43—H43C	109.5
O2—C8—O1	123.6 (2)	H43A—C43—H43C	109.5
O2—C8—C9	125.3 (2)	H43B—C43—H43C	109.5
O1—C8—C9	111.1 (2)	N3—C44—C39	99.8 (4)
C10—C9—C8	125.5 (2)	N3—C44—C46	111.7 (4)
C10—C9—C14	112.9 (2)	C39—C44—C46	113.3 (5)
C8—C9—C14	121.6 (2)	N3—C44—C45	107.8 (4)
C9—C10—C11	113.2 (2)	C39—C44—C45	112.7 (4)
C9—C10—H10A	123.4	C46—C44—C45	111.0 (4)
C11—C10—H10A	123.4	C44—C45—H45A	109.5
N1—C11—C10	99.52 (18)	C44—C45—H45B	109.5
N1—C11—C12	110.3 (2)	H45A—C45—H45B	109.5
C10—C11—C12	112.9 (2)	C44—C45—H45C	109.5
N1—C11—C13	109.2 (2)	H45A—C45—H45C	109.5
C10—C11—C13	112.6 (2)	H45B—C45—H45C	109.5
C12—C11—C13	111.6 (2)	C44—C46—H46A	109.5
C11—C12—H12A	109.5	C44—C46—H46B	109.5
C11—C12—H12B	109.5	H46A—C46—H46B	109.5
H12A—C12—H12B	109.5	C44—C46—H46C	109.5
C11—C12—H12C	109.5	H46A—C46—H46C	109.5
H12A—C12—H12C	109.5	H46B—C46—H46C	109.5
H12B—C12—H12C	109.5	O8'—C38'—O7	111.5 (4)
C11—C13—H13A	109.5	O8'—C38'—C39'	128.1 (6)
C11—C13—H13B	109.5	O7—C38'—C39'	120.4 (5)
H13A—C13—H13B	109.5	C40'—C39'—C38'	125.8 (7)
C11—C13—H13C	109.5	C40'—C39'—C44'	112.0 (6)
H13A—C13—H13C	109.5	C38'—C39'—C44'	122.2 (7)
H13B—C13—H13C	109.5	C39'—C40'—C41'	113.3 (5)
N1—C14—C9	99.35 (19)	C39'—C40'—H40B	123.4
N1—C14—C16	109.3 (2)	C41'—C40'—H40B	123.4
C9—C14—C16	114.0 (2)	N3'—C41'—C40'	101.5 (4)
N1—C14—C15	109.4 (2)	N3'—C41'—C43'	110.4 (5)
C9—C14—C15	113.0 (2)	C40'—C41'—C43'	110.9 (4)
C16—C14—C15	111.1 (2)	N3'—C41'—C42'	111.6 (5)
C14—C15—H15A	109.5	C40'—C41'—C42'	113.1 (5)
C14—C15—H15B	109.5	C43'—C41'—C42'	109.2 (5)
H15A—C15—H15B	109.5	C41'—C42'—H42D	109.5
C14—C15—H15C	109.5	C41'—C42'—H42E	109.5
H15A—C15—H15C	109.5	H42D—C42'—H42E	109.5
H15B—C15—H15C	109.5	C41'—C42'—H42F	109.5
C14—C16—H16A	109.5	H42D—C42'—H42F	109.5
C14—C16—H16B	109.5	H42E—C42'—H42F	109.5
H16A—C16—H16B	109.5	C41'—C43'—H43D	109.5
C14—C16—H16C	109.5	C41'—C43'—H43E	109.5
H16A—C16—H16C	109.5	H43D—C43'—H43E	109.5
H16B—C16—H16C	109.5	C41'—C43'—H43F	109.5
C18—C17—C22	118.0 (2)	H43D—C43'—H43F	109.5
C18—C17—C1	123.6 (2)	H43E—C43'—H43F	109.5
C22—C17—C1	118.2 (2)	C46'—C44'—N3'	111.9 (6)
C17—C18—C19	121.1 (2)	C46'—C44'—C39'	114.3 (7)
C17—C18—H18A	119.5	N3'—C44'—C39'	98.4 (5)
C19—C18—H18A	119.5	C46'—C44'—C45'	115.5 (7)
C20—C19—C18	118.8 (2)	N3'—C44'—C45'	105.6 (7)
C20—C19—H19A	120.6	C39'—C44'—C45'	109.4 (7)
C18—C19—H19A	120.6	C44'—C45'—H45D	109.5
C19—C20—C21	121.5 (2)	C44'—C45'—H45E	109.5
C19—C20—O4	120.3 (2)	H45D—C45'—H45E	109.5
C21—C20—O4	118.1 (2)	C44'—C45'—H45F	109.5
C22—C21—C20	118.7 (3)	H45D—C45'—H45F	109.5
C22—C21—H21A	120.6	H45E—C45'—H45F	109.5
C20—C21—H21A	120.6	C44'—C46'—H46D	109.5
C21—C22—C17	121.7 (3)	C44'—C46'—H46E	109.5
C21—C22—H22A	119.2	H46D—C46'—H46E	109.5
C17—C22—H22A	119.2	C44'—C46'—H46F	109.5
O5—C23—O4	124.0 (2)	H46D—C46'—H46F	109.5
O5—C23—C24	124.5 (3)	H46E—C46'—H46F	109.5
O4—C23—C24	111.4 (3)	C52—C47—C48	119.4 (3)
C25—C24—C23	125.7 (2)	C52—C47—H47A	120.3
C25—C24—C29	112.7 (2)	C48—C47—H47A	120.3
C23—C24—C29	121.5 (2)	C49—C48—C47	120.3 (3)
C24—C25—C26	113.4 (2)	C49—C48—H48A	119.8
C24—C25—H25A	123.3	C47—C48—H48A	119.8
C26—C25—H25A	123.3	C50—C49—C48	119.8 (3)
N2—C26—C25	98.93 (19)	C50—C49—H49A	120.1
N2—C26—C27	110.5 (2)	C48—C49—H49A	120.1
C25—C26—C27	113.5 (2)	C51—C50—C49	120.6 (3)
N2—C26—C28	110.1 (2)	C51—C50—H50A	119.7
C25—C26—C28	111.2 (2)	C49—C50—H50A	119.7
C27—C26—C28	111.9 (2)	C50—C51—C52	119.5 (3)
C26—C27—H27A	109.5	C50—C51—H51A	120.2
C26—C27—H27B	109.5	C52—C51—H51A	120.2
H27A—C27—H27B	109.5	C47—C52—C51	120.4 (3)
C26—C27—H27C	109.5	C47—C52—H52A	119.8
H27A—C27—H27C	109.5	C51—C52—H52A	119.8
H27B—C27—H27C	109.5	C55^i^—C53—C54	120.5 (4)
C26—C28—H28A	109.5	C55^i^—C53—H53A	119.7
C26—C28—H28B	109.5	C54—C53—H53A	119.7
H28A—C28—H28B	109.5	C53—C54—C55	120.4 (4)
C26—C28—H28C	109.5	C53—C54—H54A	119.8
H28A—C28—H28C	109.5	C55—C54—H54A	119.8
H28B—C28—H28C	109.5	C53^i^—C55—C54	119.1 (4)
N2—C29—C24	99.4 (2)	C53^i^—C55—H55A	120.5
N2—C29—C31	109.2 (2)	C54—C55—H55A	120.5
C24—C29—C31	112.9 (3)	C57—C56—C61	120.2 (5)
N2—C29—C30	109.1 (2)	C57—C56—H56A	119.9
C24—C29—C30	112.7 (2)	C61—C56—H56A	119.9
C31—C29—C30	112.7 (3)	C56—C57—C58	119.6 (5)
C29—C30—H30A	109.5	C56—C57—H57A	120.2
C29—C30—H30B	109.5	C58—C57—H57A	120.2
H30A—C30—H30B	109.5	C59—C58—C57	121.2 (5)
C29—C30—H30C	109.5	C59—C58—H58A	119.4
H30A—C30—H30C	109.5	C57—C58—H58A	119.4
H30B—C30—H30C	109.5	C58—C59—C60	119.9 (5)
C29—C31—H31A	109.5	C58—C59—H59A	120.1
C29—C31—H31B	109.5	C60—C59—H59A	120.1
H31A—C31—H31B	109.5	C59—C60—C61	121.9 (5)
C29—C31—H31C	109.5	C59—C60—H60A	119.0
H31A—C31—H31C	109.5	C61—C60—H60A	119.0
H31B—C31—H31C	109.5	C60—C61—C56	117.1 (5)
C37—C32—C33	118.2 (2)	C60—C61—H61A	121.4
C37—C32—C1	120.1 (2)	C56—C61—H61A	121.4
C33—C32—C1	121.7 (2)		
			
C32—C1—C2—C7	−177.7 (2)	C17—C1—C32—C37	−120.5 (2)
C17—C1—C2—C7	55.8 (3)	C2—C1—C32—C37	110.0 (2)
C32—C1—C2—C3	−3.0 (3)	C17—C1—C32—C33	57.6 (3)
C17—C1—C2—C3	−129.5 (2)	C2—C1—C32—C33	−71.9 (3)
C7—C2—C3—C4	1.4 (4)	C37—C32—C33—C34	0.0 (4)
C1—C2—C3—C4	−173.3 (2)	C1—C32—C33—C34	−178.1 (2)
C2—C3—C4—C5	−0.6 (4)	C32—C33—C34—C35	−0.6 (4)
C3—C4—C5—C6	0.3 (4)	C33—C34—C35—C36	0.8 (4)
C3—C4—C5—O1	173.7 (2)	C33—C34—C35—O7	179.3 (2)
C8—O1—C5—C4	118.3 (2)	C38—O7—C35—C36	99.6 (4)
C8—O1—C5—C6	−68.2 (3)	C38'—O7—C35—C36	−113.8 (5)
C4—C5—C6—C7	−0.9 (4)	C38—O7—C35—C34	−78.9 (5)
O1—C5—C6—C7	−174.1 (2)	C38'—O7—C35—C34	67.7 (5)
C3—C2—C7—C6	−2.1 (4)	C34—C35—C36—C37	−0.5 (4)
C1—C2—C7—C6	172.9 (2)	O7—C35—C36—C37	−178.9 (2)
C5—C6—C7—C2	1.9 (4)	C33—C32—C37—C36	0.3 (3)
C5—O1—C8—O2	−1.2 (3)	C1—C32—C37—C36	178.4 (2)
C5—O1—C8—C9	178.68 (19)	C35—C36—C37—C32	−0.1 (4)
O2—C8—C9—C10	179.2 (2)	C35—O7—C38—O8	−14.1 (7)
O1—C8—C9—C10	−0.7 (3)	C35—O7—C38—C39	172.1 (3)
O2—C8—C9—C14	−0.6 (4)	O7—C38—C39—C40	−4.5 (7)
O1—C8—C9—C14	179.4 (2)	O8—C38—C39—C40	−178.1 (5)
C8—C9—C10—C11	−178.5 (2)	O7—C38—C39—C44	172.8 (5)
C14—C9—C10—C11	1.4 (3)	O8—C38—C39—C44	−0.8 (8)
O3—N1—C11—C10	175.2 (2)	C38—C39—C40—C41	178.1 (5)
C14—N1—C11—C10	−0.7 (2)	C44—C39—C40—C41	0.6 (7)
O3—N1—C11—C12	−65.9 (3)	C39—C40—C41—C42	125.7 (7)
C14—N1—C11—C12	118.2 (2)	C39—C40—C41—N3	4.6 (7)
O3—N1—C11—C13	57.1 (3)	C39—C40—C41—C43	−108.7 (7)
C14—N1—C11—C13	−118.8 (2)	O9—N3—C41—C42	55.6 (9)
C9—C10—C11—N1	−0.4 (3)	C44—N3—C41—C42	−134.6 (6)
C9—C10—C11—C12	−117.3 (2)	O9—N3—C41—C40	−177.9 (5)
C9—C10—C11—C13	115.1 (2)	C44—N3—C41—C40	−8.1 (7)
O3—N1—C14—C9	−174.5 (2)	O9—N3—C41—C43	−62.0 (7)
C11—N1—C14—C9	1.4 (2)	C44—N3—C41—C43	107.9 (6)
O3—N1—C14—C16	−54.8 (3)	O9—N3—C44—C39	178.7 (5)
C11—N1—C14—C16	121.0 (2)	C41—N3—C44—C39	8.3 (6)
O3—N1—C14—C15	67.0 (3)	O9—N3—C44—C46	58.7 (7)
C11—N1—C14—C15	−117.2 (2)	C41—N3—C44—C46	−111.7 (6)
C10—C9—C14—N1	−1.6 (2)	O9—N3—C44—C45	−63.5 (6)
C8—C9—C14—N1	178.25 (19)	C41—N3—C44—C45	126.2 (5)
C10—C9—C14—C16	−117.7 (2)	C40—C39—C44—N3	−5.4 (6)
C8—C9—C14—C16	62.2 (3)	C38—C39—C44—N3	177.1 (5)
C10—C9—C14—C15	114.2 (2)	C40—C39—C44—C46	113.5 (5)
C8—C9—C14—C15	−65.9 (3)	C38—C39—C44—C46	−64.1 (7)
C32—C1—C17—C18	−92.5 (3)	C40—C39—C44—C45	−119.5 (5)
C2—C1—C17—C18	37.0 (3)	C38—C39—C44—C45	62.9 (7)
C32—C1—C17—C22	82.6 (3)	C35—O7—C38'—O8'	6.4 (7)
C2—C1—C17—C22	−148.0 (2)	C35—O7—C38'—C39'	−175.6 (4)
C22—C17—C18—C19	−2.8 (4)	O8'—C38'—C39'—C40'	−171.8 (6)
C1—C17—C18—C19	172.2 (2)	O7—C38'—C39'—C40'	10.5 (9)
C17—C18—C19—C20	0.3 (4)	O8'—C38'—C39'—C44'	10.9 (9)
C18—C19—C20—C21	2.9 (4)	O7—C38'—C39'—C44'	−166.8 (5)
C18—C19—C20—O4	−179.7 (2)	C38'—C39'—C40'—C41'	−179.1 (5)
C23—O4—C20—C19	86.6 (3)	C44'—C39'—C40'—C41'	−1.5 (7)
C23—O4—C20—C21	−95.9 (3)	O9'—N3'—C41'—C40'	−178.7 (6)
C19—C20—C21—C22	−3.3 (5)	C44'—N3'—C41'—C40'	−2.7 (7)
O4—C20—C21—C22	179.2 (3)	O9'—N3'—C41'—C43'	63.7 (8)
C20—C21—C22—C17	0.6 (5)	C44'—N3'—C41'—C43'	−120.4 (6)
C18—C17—C22—C21	2.4 (5)	O9'—N3'—C41'—C42'	−58.0 (8)
C1—C17—C22—C21	−172.9 (3)	C44'—N3'—C41'—C42'	118.0 (6)
C20—O4—C23—O5	−6.1 (4)	C39'—C40'—C41'—N3'	2.6 (6)
C20—O4—C23—C24	172.3 (2)	C39'—C40'—C41'—C43'	119.8 (5)
O5—C23—C24—C25	−179.2 (3)	C39'—C40'—C41'—C42'	−117.1 (6)
O4—C23—C24—C25	2.4 (4)	O9'—N3'—C44'—C46'	−61.3 (9)
O5—C23—C24—C29	2.6 (4)	C41'—N3'—C44'—C46'	122.5 (7)
O4—C23—C24—C29	−175.8 (2)	O9'—N3'—C44'—C39'	178.1 (6)
C23—C24—C25—C26	−177.2 (2)	C41'—N3'—C44'—C39'	1.9 (7)
C29—C24—C25—C26	1.2 (3)	O9'—N3'—C44'—C45'	65.1 (8)
O6—N2—C26—C25	177.9 (2)	C41'—N3'—C44'—C45'	−111.0 (7)
C29—N2—C26—C25	1.7 (3)	C40'—C39'—C44'—C46'	−118.9 (7)
O6—N2—C26—C27	−62.8 (3)	C38'—C39'—C44'—C46'	58.7 (9)
C29—N2—C26—C27	121.0 (2)	C40'—C39'—C44'—N3'	−0.2 (7)
O6—N2—C26—C28	61.3 (3)	C38'—C39'—C44'—N3'	177.5 (5)
C29—N2—C26—C28	−114.9 (2)	C40'—C39'—C44'—C45'	109.7 (7)
C24—C25—C26—N2	−1.7 (3)	C38'—C39'—C44'—C45'	−72.6 (8)
C24—C25—C26—C27	−118.7 (3)	C52—C47—C48—C49	0.0 (4)
C24—C25—C26—C28	114.0 (3)	C47—C48—C49—C50	−0.2 (4)
O6—N2—C29—C24	−177.2 (2)	C48—C49—C50—C51	0.4 (4)
C26—N2—C29—C24	−1.1 (3)	C49—C50—C51—C52	−0.4 (5)
O6—N2—C29—C31	−58.9 (3)	C48—C47—C52—C51	0.0 (5)
C26—N2—C29—C31	117.2 (3)	C50—C51—C52—C47	0.2 (5)
O6—N2—C29—C30	64.7 (3)	C55^i^—C53—C54—C55	0.3 (6)
C26—N2—C29—C30	−119.2 (2)	C53—C54—C55—C53^i^	−0.3 (6)
C25—C24—C29—N2	−0.1 (3)	C61—C56—C57—C58	−3.3 (7)
C23—C24—C29—N2	178.4 (2)	C56—C57—C58—C59	0.9 (7)
C25—C24—C29—C31	−115.7 (3)	C57—C58—C59—C60	1.2 (7)
C23—C24—C29—C31	62.8 (3)	C58—C59—C60—C61	−0.9 (8)
C25—C24—C29—C30	115.3 (3)	C59—C60—C61—C56	−1.4 (8)
C23—C24—C29—C30	−66.3 (3)	C57—C56—C61—C60	3.5 (7)

Symmetry codes: (i) −*x*+2, −*y*+1, −*z*+1.

### Hydrogen-bond geometry (Å, °)

**Table:**

Cg1, Cg2, and Cg3 are the centroids of the C47–C52, C17–C22 and C56–C61 rings, respectively.

**Table:**

*D*—H···*A*	*D*—H	H···*A*	*D*···*A*	*D*—H···*A*
C1—H1A···O6^ii^	1.00	2.49	3.448 (3)	160
C4—H4A···O9'^iii^	0.95	2.44	3.165 (5)	133
C25—H25A···O2^iv^	0.95	2.57	3.358 (4)	141
C37—H37A···O6^ii^	0.95	2.57	3.442 (3)	153
C57—H57A···O6^v^	0.95	2.58	3.514 (8)	168
C10—H10A···Cg1	0.95	2.84	3.752 (3)	161
C12—H12A···Cg2^v^	0.98	2.94	3.726 (3)	138
C19—H19A···Cg1^vi^	0.95	2.79	3.725 (3)	169
C34—H34A···Cg3	0.95	2.78	3.547 (4)	138

Symmetry codes: (ii) *x*−1, *y*−1, *z*; (iii) −*x*+1, −*y*, −*z*; (iv) *x*, *y*+1, *z*; (v) *x*, *y*−1, *z*;  (vi) *x*+1, *y*+1, *z*.
